# Comparison of Efficacy between Clopidogrel and Ticagrelor in Patients with Acute Coronary Syndrome after Interventional Treatment and Their Effects on IL-6

**Published:** 2020-02

**Authors:** Bin YANG, Chunyan ZHENG, Haichu YU, Rui ZHANG, Shan LI, Min LEN, Shanglang CAI

**Affiliations:** 1. Department of Cardiology, The Affiliated Hospital of Qingdao University, Qingdao, China; 2. Medical Consultation Center, The Affiliated Hospital of Qingdao University, Qingdao, China

**Keywords:** Clopidogrel, Ticagrelor, Acute coronary syndrome, Interleukin-6, Platelet aggregation

## Abstract

**Background:**

We aimed to compare the efficacy between clopidogrel and ticagrelor in patients with acute coronary syndrome (ACS) after percutaneous coronary intervention (PCI) and their effects on IL-6.

**Methods:**

A retrospective analysis and collection of 200 ACS patients diagnosed by the Department of Cardiology, Affiliated Hospital of Qingdao University, Qingdao, China in 2016 were performed. Patients were randomly divided into clopidogrel group and ticagrelor group. Data of left ventricular ejection fraction and ACS clinical classification before PCI, PCI treatment, IL-6, platelet aggregation status, maximum platelet aggregation rate (MPAR), P2Y12 response unit (PRU) and adverse reaction of patients were collected. After PCI, patients were followed up for 1 year to compare the ischemia after treatment between clopidogrel group and tigravilol group.

**Results:**

MPAR and PRU after PCI of clopidogrel group were significantly higher than those of ticagrelor group (*P*<0.05). The expression of IL-6 in two groups peaked at 1 day after PCI and then decreased. That of ticagrelor group was consistently lower than that of clopidogrel group (*P*<0.05). The incidence of ischemic events after treatment in clopidogrel group was significantly higher than that in ticagrelor group (*P*<0.001).

**Conclusion:**

Compared with clopidogrel, tigerrilol had more significant inhibition of platelet aggregation after PCI in ACS patients, and tigerrilol had better effect after interventional treatment in ACS patients. In addition, compared with clopidogrel, tegrel can significantly inhibit the expression of IL-6 in patients with ACS and better alleviate the inflammatory response after PCI.

## Introduction

As a cardiovascular disease with an extremely high incidence, acute coronary Commented [A2]: Separate the paragraphs syndrome (ACS) (1–3) belongs to coronary heart disease. It is mainly caused by the high aggregation of platelet in the patient’s myocardium and the intraluminal thrombus resulting in sudden myocardial ischemia or insufficient blood supply. Its pathophysiological mechanism is the coronary plaque rupture stimulated by acute inflammation (4,5).

Acting as an inflammatory factor, interleukin-6 (IL-6) in serum can promote inflammatory response in myocardial cells (6). The inflammatory response induced by IL-6 leads to myocardial damage and dysfunction in various causes. This is closely related to the occurrence and development of ACS (7). For the treatment of it (8), percutaneous coronary intervention (PCI) is generally used clinically (9). PCI that is very effective in the treatment of ACS patients can slowly reduce all aspects of adverse symptoms in them, so that the mortality of them has greatly reduced. However, ACS is a chronic acute and severe disease, so patients should take relevant drugs after PCI according to the doctor’s advice. For ACS patients after PCI, anti-platelet drugs (10) are particularly important, among which, clopidogrel (11) and ticagrelor (12) are commonly used in them. Both clopidogrel and ticagrelor can alleviate and treat platelet aggregation. At present, a large amount of data (13–15) show that aspirin combined with clopidogrel for ACS patients after PCI has an excellent effect on anti-platelet aggregation. However, since clopidogrel is a pro-drug, its transformation in different patients is different in size, leading to the instability of its therapeutic effect. Studies have shown (16) that some ACS patients are intolerant or resistant to clopidogrel, which increases the risk of taking it. Ticagrelor is also an inhibitor of platelet aggregation (12,17). It may also have adverse reactions such as dyspnea and slow arrhythmia in some ACS patients.

In this study, the efficacy evaluation between clopidogrel and ticagrelor in ACS patients after interventional treatment and their effects on IL-6 in the serum of ACS patients were compared.

## Methods

### Information collection

A retrospective analysis and collection of 200 ACS patients diagnosed by the Department of Cardiology, Affiliated Hospital of Qingdao University, China from January 2016 to December 2016 were performed, including 112 male patients and 88 female patients, aged from 45 to 80 years old, with an average range of (59.38±9.74) years old. They were randomly divided into clopidogrel group (100 cases) and ticagrelor group (100 cases).

This study was approved by Affiliated Hospital of Qingdao University. The informed consent was signed by the participants before the study.

### Inclusion and exclusion criteria

1) Only patients treated in The Affiliated Hospital of Qingdao University who met the international ACS diagnostic guideline were included.
2) Patients with immune system diseases, family genetic diseases and various tumors and cancers were excluded; patients with liver or kidney dysfunction and previous coagulopathy excluded; pregnant women excluded. All included patients and their family members signed the informed form.

### Main reagents, instruments and drugs

IL-6 enzyme-linked immunoassay kit (Nanjing Senbega Biotechnology), PL-12 platelet aggregation analyzer and its supporting reagents (Jiangsu Yingnuohua Medical Technology Co., Ltd.), Verify Now anti-platelet therapy monitoring system and its supporting reagents (Jiangsu Yingnuohua Medical Technology Co., Ltd.), clopidogrel (SFDA approval number H20000542), aspirin (SFDA approval number J20130053) and ticagrelor (SFDA approval number J201300200) were purchased from Lepu Pharmaceutical Co., Ltd..

### Treatment of subjects and methods of specimen collection

1) In clopidogrel group, 300 mg of load quantity aspirin and 300 mg of clopidogrel were administered orally 30 min before PCI, and 100 mg of aspirin once daily and 75 mg of clopidogrel once daily administered orally after PCI. In ticagrelor group, 300 mg of load quantity aspirin and 180 mg of ticagrelor were administered orally before PCI, and 100 mg of aspirin once daily and 90 mg of ticagrelor twice daily administered orally after PCI. Data of left ventricular ejection fraction and ACS clinical classification 30 min before PCI, PCI treatment, IL-6 and platelet aggregation status at different time points before PCI (T0), at 1 day after PCI (T1), 7 days after PCI (T2) and 30 days after PCI (T3) of patients were collected.
2) Sodium citrate anticoagulation vacuum blood collection tube was used to collect elbow venous blood (4 tubes at each time interval, 2 mL per tube) from fasting ACS patients at different time points before PCI (T0), at 1 day after PCI (T1), 7 days after PCI (T2) and 30 days after PCI (T3). Maximum platelet aggregation rate (MPAR), P2Y12 reaction unit (PRU), and IL-6 concentration were detected. All specimens were subjected to cryogenic centrifugation within 3 hours (4°C, 3500 r/min, 15 min), placed in a -80°C refrigerator for testing.
3) MPAR was determined using PL-12 platelet aggregation analyzer and its supporting reagents. The specimen was taken at the position to be tested of the instrument, the button pressed to test and the result printed. PRU testing was determined using Verify Now anti-platelet therapy monitoring system and its supporting reagents, IL-6 using enzyme-linked immunoassay. Centrifugation at 3500 r/min was performed to isolate serum which was then placed - 20 °C low temperature refrigerator. The test was conducted strictly according to the operating instructions of IL-6 ELISA test kit. 100 μL standard liquid, sample to be tested, and negative and positive control liquid were taken to the reaction hole. 100 μL biological reaction anti-body fluid was added rapidly. Cover with a membrane, and leave for 40 min after mixing. Then 100 μL of streptomycin was added to each reaction hole. Cover with a membrane, and then leave for 40 min after blending. Pour out the liquid in the reaction hole, add cleaning liquid to each reaction hole, slowly shake the mixer for 1 min, pour out the liquid in the reaction hole, and repeat 5 times. 100 μL of reaction substrate A and 100 μL of reaction substrate B were added to each reaction hole, covered with a membrane, and left in the dark for 5 min. 100 μL terminated liquid was added to the reaction hole, and OD value of each hole was measured at the wavelength of 450 nm by using the enzyme standard analyzer, then IL-6 level was calculated.

### Statistical methods

SPSS19.0 statistical software (Chicago, IL, USA) was used for the analysis of statistical data. Measurement data were expressed as (x̄±s). χ^2^ test was used for the comparison of count data between groups. Paired t test was used for pairwise comparison between groups at different time points or between groups, and one-way ANOVA test was used for comparison at multiple time points within the group. When *P*<0.05, the difference is statistically significant.

## Results

### Clinical baseline information of clopidogrel group and ticagrelor group

The general clinical baseline information of ACS patients before PCI between clopidogrel group and ticagrelor group were compared. The results showed that the data difference was not statistically significant between two groups in age, left ventricular ejection fraction, ACS clinical classification, whether statin drugs affecting IL-6 levels were used before treatment, PCI treatment, body mass index, total cholesterol, triglyceride, systolic blood pressure and diastolic blood pressure (Table 1).

**Table 1:** Clinical baseline information of clopidogrel group and ticagrelor group

***Groups***		***Clopidogrel group (n=100)***	***Ticagrelor group (n=100)***	***t/x^2^***	**P**
Male [n, (%)]		55 (55.00)	57 (57.00)	0.080	0.777
Age (yr)		59.34±9.25	60.56±8.75	0.715	0.476
Body mass index (kg/m^2^)		25.89±1.45	25.76±1.26	0.677	0.499
Total cholesterol (mnol·L^−1^)		7.32±1.34	7.29±1.60	0.144	0.886
Triglyceride (mnol·L^−1^)		2.05±1.12	1.99±0.97	0.405	0.686
				0	
Systolic blood pressure (mmHg)		137.62±15.97	135.89±15.08	0.788	0.432
Diastolic blood pressure (mmHg)		95.56±10.05	94.04±10.34	1.054	0.293
Left ventricular ejection fraction (%)		50.89±4.57	51.09±5.56	0.320	0.750
ACS clinical	STEMI	19 (19.00)	15 (15.00)	0.567	0.452
classification	NSTEMI	24 (24.00)	34 (34.00)	2.428	0.119
(18) [n, (%)]	AUAP	57 (57.00)	51 (51.00)	0.725	0.395
PCI treatment (mm)	Maximum stent length	23.98±6.45	24.03±6.27	0.056	0.956
	Minimum stent length	18.86±3.65	19.16±2.89	0.859	0.391
	Maximum stent diameter	3.45±1.27	3.46±1.84	0.045	0.964
	Minimum stent diameter	2.90±0.68	2.82±1.34	0.532	0.595
Statins were used before treatment	Yes	0(0.00)	0(0.00)	0.000	1.000
	No	100(100.00)	100(100.00)	0.000	1.000

### Comparison of platelet aggregation function between clopidogrel group and ticagrelor group

The difference was not statistically significant in MPAR and PRU of ACS patients at the time of T0 between two groups. MPAR at the time of T1, T2 and T3 in clopidogrel group was significantly higher than that in ticagrelor group, with statistically significant differences (Pt1=0.002, Pt2=2.680*10-6, Pt3=2.021*10-10). PRU at the time of T1, T2 and T3 in clopidogrel group were significantly higher than that in ticagrelor group, with statistically significant differences (Pt1=0.002, Pt2=2.680*10-6, Pt3=2.021*10-10) (Tables 2, 3).

**Table 2:** Comparison of MPAR between clopidogrel group and ticagrelor group

***Groups***		***Clopidogrel group (n=100)***	***Ticagrelor group (n=100)***	**t**	**P**
MPAR (%)	T0	61.75±12.49	60.89±13.35	0.522	0.602
	T1	61.66±13.73	55.71±12.49^*^	3.612	0.002
	T2	49.20±9.67^*#^	42.36±10.33^*#^	4.152	2.680^*^10^−6^
	T3	39.78±10.73^*#Δ^	30.14±9.56^*#Δ^	5.852	2.021^*^10^−10^
F		49.971	219.844		
*P*		2.4823^*^10^−41^	4.8294^*^10^−63^		

Note: The measurement data in the table are expressed as (X±S). Pairwise t-test is used to compare between two different time points within the group or between groups. The comparison of multiple time points within the group is analyzed by repeated measures analysis of variance followed by Bonferroni test, represented by F. When *P* < 0.05, the difference is statistically significant.

* *P* < 0.05, vs. T0;

# *P* < 0.05, vs. T1;

Δ P < 0.05, vs. T2

**Table 3:** Comparison of PRU (U) between clopidogrel group and ticagrelor group

***Groups***	***Clopidogrel group (n=100)***	***Ticagrelor group (n=100)***	**t**	**P**
T0	250.77±15.86	249.38±16.04	0.700	0.484
T1	223.89±14.61^*^	213.54±14.87^*^	5.215	1.480^*^10^−6^
PRU (U)	210.75±13.94^*^	190.86±12.50^*#^	10.023	1.888^*^10^−21^
T2				
T3	144.95±12.73^*#Δ^	115.25±10.92^*#Δ^	14.966	5.554^*^10^−43^
F	238.777	2622.001		
*P*	3.8220^*^10^−183^	5.0423^*^10^−226^		

Note: The measurement data in the table are expressed as (X±S). Pairwise t-test is used to compare between two different time points within the group or between groups. The comparison of multiple time points within the group is analyzed by repeated measures analysis of variance followed by Bonferroni test, represented by F. When *P* < 0.05, the difference is statistically significant.

* *P* < 0.05, vs. T0;

# *P* < 0.05, vs. T1;

Δ P < 0.05, vs. T2

### Comparison of expression of IL-6 before and after treatment between clopidogrel group and ticagrelor group

**Fig. 1:**
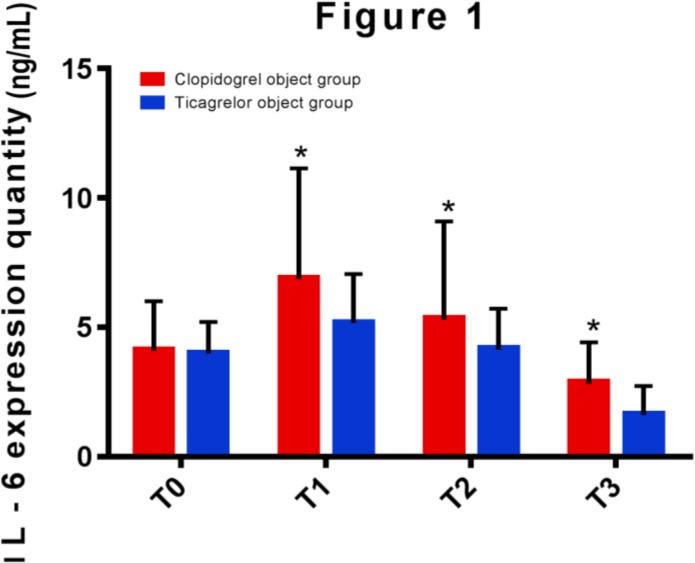
Detection of expression of IL-6 before PCI in clopidogrel group and ticagrelor group by enzyme-linked immunosorbent assay. (Note: * indicates that the data of this group is significantly higher than that of the other group, with a statistically significant difference between two groups)

The expression of IL-6 before PCI (at T0) in clopidogrel group and ticagrelor group were (4.12±1.89) ng/mL and (4.01±1.20) ng/mL, respectively, and the difference was not statistically significant. Due to the inflammatory response after PCI, that of IL-6 peaked at T1 and then gradually decreased. Those of IL-6 at T1, T2 and T3 in ticagrelor group were (5.17±1.88) ng/mL, (4.18±1.54) ng/mL and (1.66±1.07) ng/mL, respectively, significantly lower than those in clopidogrel group, which were (6.89±4.25) ng/mL, (5.34±3.76) ng/mL and (2.87±1.55) ng/mL, respectively. The difference was statistically significant between two groups of data (*P*<0.05) (Fig. 1).

### Comparison of follow-up after treatment between clopidogrel group and ticagrelor group

One-year follow-up of patients after PCI was performed to compare the ischemic status of clopidogrel group and ticagrelor group after treatment. The follow-up data showed that the incidence of ischemic events after treatment in clopidogrel group was 32.00%, significantly higher than that in ticagrelor group (4.00%), with a statistically significant difference (*P*<0.001) (Tables 4, 5).

**Table 4:** Comparison of IL-6 (ng/mL) between clopidogrel group and ticagrelor group

***Groups***		***Clopidogrel group (n=100)***	***Ticagrelor group (n=100)***	**t**	**P**
IL-6 (ng/mL)	T0	4.12±1.89	4.01±1.20	0.332	0.748
	T1	6.89±4.25^*^	5.17±1.88^*^	3.701	0.001
	T2	5.34±3.76^*^	4.18±1.54^*#^	2.855	0.005
	T3	2.87±1.55^*#Δ^	1.66±1.07^*#Δ^	6.424	9.645^*^10^−10^
F		37.801	84.566		
*P*		5.7364^*^10^−18^	4.0025^*^10^−42^		

Note: The measurement data in the table are expressed as (X±S). Pairwise t-test is used to compare between two different time points within the group or between groups. The comparison of multiple time points within the group is analyzed by repeated measures analysis of variance followed by Bonferroni test, represented by F. When *P* < 0.05, the difference is statistically significant.

* *P* < 0.05, vs. T0;

# *P* < 0.05, vs. T1;

Δ P < 0.05, vs. T2

**Table 5:** Comparison of ischemic status after treatment between clopidogrel group and ticagrelor group

***Groups***		***Clopidogrel group (n=100)***	***Ticagrelor group (n=100)***	***X^2^***	**P**
Ischemic events [n (%)]	In-stent restenosis	16 (16.00)	2 (2.00)	11.97	<0.001
	Cerebral thrombosis	8 (8.00)	2 (2.00)	3.812	0.051
	Sudden cardiac death	4 (4.00)	0 (0.00)	4.082	0.043
	Sum	32 (32.00)	4 (4.00)	26.56	<0.001

Note: The count data in the table is tested by χ2. When *P*<0.05, the difference is statistically significant

## Discussion

The biggest feature of ACS patients (19) is arterial blockage. When blood vessels are blocked, the general clinical operative plan is to clear them. The preferred option is PCI treatment. As a minimally invasive operation, the principle of PCI is to open a tiny channel in the patient’s brachial artery using minimally invasive puncture technique. Then a specific guide wire and catheter are introduced into the channel, extending to the patient’s coronary arteries of cardiovascular system.

Finally, a contrast agent for contrast is put into. So that, the doctor can perform dredge or stent implantation based on the cardiovascular obstruction of ACS patients, so as to achieve the treatment technique changing the size of blood flow in the myocardium (20).

PCI, the primary treatment method for ACS patients, is very effective in the treatment of ACS. However, from the long-term perspective, patients will suffer mechanical damage to the endangium due to the stent implantation during PCI operation. As a foreign material, the sustained stimulation of postoperative stent causes the platelet and inflammatory cells to aggregate, releasing the inflammatory mediator IL-6, thereby enhancing the expression of IL-6. As a result, the inflammatory response of blood vessels is enhanced to some extent (21). IL-6 can promote structural restenosis and platelet re-aggregation in PCI-implanted stent (22). Therefore, it is especially important to take the corresponding anti-platelet aggregation or inflammation-inhibiting drugs according to the doctor’s advice. In this study, the efficacy between commonly used clinically clopidogrel and ticagrelor in ACS patients after interventional treatment and their effects on IL-6 in the serum of patients were compared.

In this study, ACS patients’ age, left ventricular ejection fraction, ACS clinical classification, PCI treatment, body mass index, total cholesterol, triglyceride, systolic blood pressure and diastolic blood pressure before PCI in clopidogrel group and ticagrelor group were compared. The results showed that the data difference was not statistically significant in the clinical baseline between two groups, which reduced the deviation of detection results to some extent.

Firstly, MPAR and PRU in clopidogrel group at different time points were compared at 1 day, 7 days and 30 days after PCI. Both MPAR and PRU showed a downward trend, with a statistically significant difference between groups (all *P*<0.001). MPAR and PRU in ticagrelor group were also compared similarly. Both of them showed a downward trend, with a statistically significant difference between groups (all *P*<0.001). Then, the platelet aggregation function between clopidogrel group and ticagrelor group were compared. Both MPAR and PRU of patients in clopidogrel group were significantly higher than those in ticagrelor group at 1 day, 7 days and 30 days after PCI, with a statistically significant difference (all *P*<0.05).

Therefore, it is speculated that after taking ticagrelor, the effect of lowering platelet aggregation rate was better than that of patients taking clopidogrel. Patients taking ticagrelor had a significantly lower platelet aggregation rate than those taking clopidogrel at different time points after PCI (23). This is consistent with the point of view of this article. After that, the expression of IL-6 before and after treatment between clopidogrel group and ticagrelor group were compared. The expressions of IL-6 in ticagrelor group of patients at 1 day, 7 days and 30 days after PCI were significantly lower than those in clopidogrel group (all *P*<0.05), similar to another study (24). The expression of IL-6 in the serum of ACS patients after PCI taking clopidogrel and ticagrelor was observed by them. The results showed that that of IL-6 in ticagrelor group of patients after PCI was significantly lower than that in clopidogrel group (all *P*<0.05).

Finally, in order to observe possible adverse reactions after treatment in clopidogrel group and ticagrelor group, one-year follow-up of patients after PCI was performed to compare the ischemic status of two groups after treatment. The follow-up data showed that the incidence of ischemic events after treatment in clopidogrel group was 32.00%, significantly higher than that in ticagrelor group (4.00%)(*P*<0.001). Some ACS patients have resistance to clopidogrel, often causing instent re-thrombosis, cerebral thrombosis and acute myocardial infarction (25).

In this study, the experimental bias caused by the difference in clinical baseline data of subjects was reduced. However, due to the insufficient number of included subjects and the lack of long-term follow-up, some results may have certain contingency. To better improve study results, regular follow-up will be performed on subjects.

## Conclusion

Compared to clopidogrel, ticagrelor is more effective in inhibiting platelet aggregation in ACS patients after PCI, having a better efficacy in ACS patients after interventional treatment. In addition, it can more significantly inhibit the expression of IL-6 in ACS patients, better alleviating the inflammatory response after PCI.

## Ethical considerations

Ethical issues (Including plagiarism, informed consent, misconduct, data fabrication and/or falsification, double publication and/or submission, redundancy, etc.) have been completely observed by the authors.
